# The Role of Natural Compounds and their Nanocarriers in the Treatment of CNS Inflammation

**DOI:** 10.3390/biom10101401

**Published:** 2020-10-01

**Authors:** Bikram Khadka, Jae-Young Lee, Dong Ho Park, Ki-Taek Kim, Jong-Sup Bae

**Affiliations:** 1 Department of Biomedicine, Health & Life Convergence Sciences, BK21 Four, Mokpo National University, Muan-gun, Jeonnam 58554, Korea; khadkabikram180@gmail.com; 2 College of Pharmacy, Chungnam National University, Daejeon 34134, Korea; jaeyoung@cnu.ac.kr; 3 Department of Ophthalmology, School of Medicine, Kyungpook National University, Kyungpook National University Hospital, Daegu 41944, Korea; DongHo_Park@knu.ac.kr; 4 College of Pharmacy and Natural Medicine Research Institute, Mokpo National University, Muan-gun, Jeonnam 58554, Korea; 5 College of Pharmacy, CMR1, Research Institute of Pharmaceutical Sciences, Kyungpook National University, Daegu 41566, Korea

**Keywords:** neuroinflammation, central nervous system (CNS) degenerative diseases, natural compounds, neuroprotective, nanocarriers, blood–brain barrier (BBB), targeting

## Abstract

Neuroinflammation, which is involved in various inflammatory cascades in nervous tissues, can result in persistent and chronic apoptotic neuronal cell death and programmed cell death, triggering various degenerative disorders of the central nervous system (CNS). The neuroprotective effects of natural compounds against neuroinflammation are mainly mediated by their antioxidant, anti-inflammatory, and antiapoptotic properties that specifically promote or inhibit various molecular signal transduction pathways. However, natural compounds have several limitations, such as their pharmacokinetic properties and stability, which hinder their clinical development and use as medicines. This review discusses the molecular mechanisms of neuroinflammation and degenerative diseases of CNS. In addition, it emphasizes potential natural compounds and their promising nanocarriers for overcoming their limitations in the treatment of neuroinflammation. Moreover, recent promising CNS inflammation-targeted nanocarrier systems implementing lesion site-specific active targeting strategies for CNS inflammation are also discussed.

## 1. Introduction

Although various central nervous system (CNS) disorders, particularly CNS degenerative diseases including Alzheimer’s disease (AD), Parkinson’s disease (PD), Huntington’s disease (HD), multiple sclerosis (MS), and amyotrophic lateral sclerosis (ALS), have become a bigger burden to individuals, families, and society, the molecular mechanisms and microenvironments of these disorders have not been fully understood. Neuroinflammation, which involves inflammatory cascades in nervous tissues, may be initiated in response to microglia/astrocyte activation, oxidative stress caused by reactive oxygen/nitrogen species (ROS/RNS), and mitochondrial excitotoxicity [1,2,3,4,5]. Sustained and prolonged neuroinflammation can result in persistent and chronic apoptotic neuronal cell death and programmed cell death (pyroptosis and necroptosis), thereby triggering various CNS degenerative disorders [2,6,7].

Numerous studies have demonstrated that the beneficial effects of natural compounds—derived from plants, vegetables, fruits, dietary nutrients, and endogenous molecules—on neuroinflammation were exerted owing to their antioxidant, anti-inflammatory, antiapoptotic, and neuroprotective effects [8,9]. The advantages of many natural compounds in neuroinflammation involve their high affinity for various receptors in the brain, thereby specifically promoting or inhibiting various molecular signal transduction pathways and their multi-targeting effects on various CNS disorders as well as their lower side effects compared to conventional synthetic drugs [9,10,11,12,13,14]. However, their pharmacological effects on neuroinflammation are often impeded because of their instability, poor solubility, and/or poor blood–brain barrier (BBB) permeability, resulting in lower bioavailability (BA), lower distribution in target tissue (brain), and higher systemic toxicities [15,16].

To overcome these limitations and improve their pharmacokinetic properties and stability, the role of various nanocarriers (e.g., polymeric nanoparticles (NPs), micelles, lipid NPs, liposomes, inorganic NPs, exosomes, and carbon-based NPs) have been emphasized recently, particularly in clinical trials. These nanocarriers can encapsulate or adsorb natural compounds efficiently (nanomedicines) and improve their BA, transport across the BBB, and target lesion sites in the brain, thereby enhancing their therapeutic efficacy for various neuroinflammation-induced CNS diseases [17,18]. 

In this review, we aim to introduce the molecular mechanisms of neuroinflammation and CNS degenerative diseases. In addition, we focus on potential natural compounds and the use of their promising nanocarriers to overcome their limitations in the treatment of neuroinflammation. Moreover, we discuss recent advances in therapeutic strategies for specifically targeting CNS inflammation as well as the limitations in clinical trials.

## 2. Neuroinflammation and CNS Degenerative Diseases 

Neuroinflammation refers to inflammatory processes in CNS tissues. The severity and duration of neuroinflammation depend on various stimuli and stresses to the CNS [19]. Acute neuroinflammation is favorable to the CNS, promoting clearance of the injured cells by activating the innate immune system, such as macrophages and microglia, thereby inhibiting the expansion of lesion sites [20,21]. In CNS disorders, this balance between inflammation and intrinsic neurotrophic pathways influences neurological recovery. However, prolonged and chronic inflammatory responses in the CNS lead to the enhanced release of inflammatory mediators and oxidative stress, thereby perpetuating neuroinflammation cascades and accelerating various types of neuronal cell death, which may be a crucial cause of CNS degenerative diseases [20,21]. Therefore, we need to better understand the molecular mechanisms of neuroinflammation to seek potential target molecules and determine whether the inflammatory responses are harmful or helpful.

### 2.1. Molecular Mechanisms of Neuroinflammation and Neurodegeneration

#### 2.1.1. Reactive Microglia and Astrocytes 

The activation of microglia and astrocytes is one of the factors that induce neuroinflammation. The activation of glial cells from a resting state is stimulated by pro-inflammatory stimuli, such as damage-associated molecular proteins (DAMPs) and several signaling pathways such as nuclear factor-kappa B (NF-κB) and Janus kinase (JAK)/signal transducer and activator of transcription (STAT) pathways [22]. Microglial activation can be subdivided into M1 and M2 states. M1 microglia, which are pro-inflammatory cells, release inflammatory molecules such as ROS, RNS, and pro-inflammatory cytokines, and upregulate antigen proteins such as major histocompatibility complex class II [4,23]. In contrast, M2 microglia exhibit anti-inflammatory properties including release of anti-inflammatory cytokines such as interleukin 4 (IL-4), IL-10, and transforming growth factor-beta (TGF-β), leading to tissue repair [4,23].

Particularly, reactive astrocytes can release glutamate, inhibit glutamate reuptake, and upregulate *N*-methyl-d-aspartate receptor (NMDAR) expression, leading to glutamate/NMDA/Ca^2+^ signaling-induced toxicity [24]. Similar to microglia, reactive astrocytes can be categorized into A1 and A2 states. A1 astrocytes exhibit pro-inflammatory and neurotoxic responses, whereas A2 astrocytes show neuroprotective effects [23].

#### 2.1.2. Mitochondrial Dysfunction

Mitochondrial excitotoxicity is induced by the excessive release of neurotransmitters such as glutamate from presynapse to postsynapse followed by activation of the *N*-methyl-d-aspartate (NMDA) and α-amino-3-hydroxy-5-methyl-4-isoxazole propionate (AMPA) receptors [3,25]. The over-activation of these receptors can promote an influx of extracellular Ca^2+^ ion, thereby increasing intracellular Ca^2+^ ion levels. The accumulation of a Ca^2+^ ion can affect the mitochondrial function by generating nitrogen oxide (NO) and ROS, opening the mitochondrial permeability transition pore (mPTP), and inhibiting adenosine triphosphate (ATP) production, resulting in mitochondrial dysfunction [24,25]. In addition, mPTP opening can cause the release of cytochrome c, which is a proapoptotic protein, into the cytosol, resulting in the formation of the apoptosome complex and the activation of caspase-3 and -9-based apoptosis cascade [25,26]. 

#### 2.1.3. BBB Disruption

**Figure 1:**
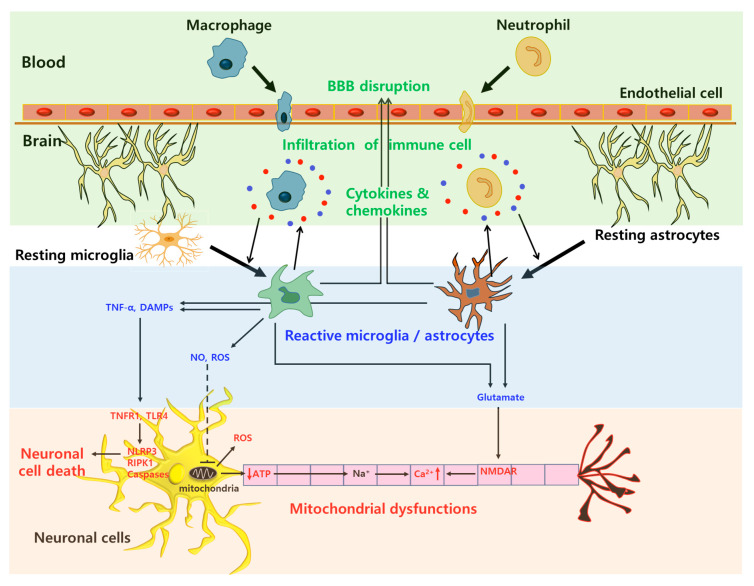
Schematic illustration of neuroinflammation processes by immune cell infiltration, reactive glial cells, and mitochondrial excitotoxicity. Blood–brain barrier (BBB); Tumor necrosis factor-α (TNF-α); Damage-associated molecular patterns (DAMPs); Nitrogen oxide (NO); Reactive oxygen species (ROS); TNF receptor 1 (TNFR1); Toll-like receptor 4 (TLR4); Nucleotide-binding oligomerization domain (NOD)-like receptor (NLR) pyrin domain 3 (NLRP3); Receptor-interacting protein kinase-1 (RIPK1); Adenosine triphosphate (ATP); N-methyl-D-aspartate receptor (NMDAR).

The blood–brain barrier (BBB) consists of endothelial cells with a higher expression of tight junctions, astrocytes, and pericytes, which cover the brain capillary lumen. The physiological barrier function of the BBB is mainly due to the tight junctions between the interconnected endothelial cells [27,28]. The breakdown of the BBB during neuroinflammation, which is mainly based on the disruption of tight junctions, allows for the infiltration of inflammatory cells such as circulating peripheral macrophages, neutrophils, and leukocytes, resulting in the generation of ROS and the activation of pro-inflammatory cytokines (IL-1β, IL-6, and TNF-α) and chemokines (Monocyte chemoattractant protein-1; MCP-1 and IL-8) into the brain parenchyma and neuronal cells [27,29,30]. Thus, the BBB disruption further accelerates neuroinflammation processes, resulting in the promotion of programmed cell death and the degeneration of neuronal axons. A schematic illustration of the various inflammation processes on neuronal cells by reactive glial cells, mitochondrial excitotoxicity, and immune cell infiltration is described in Figure 1.

#### 2.1.4. Neuronal Apoptosis

Apoptotic neuronal cell death can occur through the intrinsic and extrinsic pathways. Intrinsic apoptosis can be activated by intracellular ROS and mitochondrial dysfunctions, followed by the release of cytochrome c. The released cytochrome c forms the apoptosome complex, where pro-caspase-9 is cleaved and activated to caspase-9. Then, the caspase-9 can activate caspase-3, -6, and -7, resulting in cell death [7,31]. In addition, extrinsic apoptosis can be activated by tumor necrosis factor (TNF), TNF receptor 1 (TNFR1), and Fas (CD95), resulting in the formation of complexes containing Fas-associated death domain proteins (FADD) and pro-caspase-8, followed by caspase-8 activation and caspase-3, -6, and -7 cascades [7,31]. These apoptotic processes are considered as programmed cell death, which does not always cause inflammation, whereas other types of cell deaths, such as pyroptosis and necroptosis, accompany the inflammatory responses.

#### 2.1.5. Pyroptosis

In recent studies, non-apoptotic inflammatory cell death, such as pyroptosis and necroptosis, has emerged as a crucial contributor to neurodegeneration. Pyroptosis, a form of inflammatory regulated cell death, involves inflammasomes, the caspase-1 family (caspase-1/4/5/11), IL-1β, IL-18, and gasdermin D [27,32]. Pathogen-associated molecular patterns (PAMP) and pro-inflammatory cytokines can initiate the expression of nucleotide-binding oligomerization domain (NOD)-like receptor (NLR) pyrin domain 3 (NLRP3) and pro-1β via the Toll-like receptor 4 (TLR4)/NF-κB pathway. In the activation step, mitochondrial dysfunction, oxidative stress from ROS, an influx of Ca^2+^ ion, and DAMPs can induce the formation of the NLRP3 inflammasome complex with apoptosis-associated speck-like proteins (ASC) and caspase-1. NLRP1 inflammasomes can be activated by mitochondrial ATP depletion, an influx of Ca^2+^ ion, and extracellular amyloid β protein (Aβ) [7,33]. Then, the NLRP-based inflammasome complexes cleave pro-IL-1β and pro-IL-18 to IL-1β and IL-18. It can also activate gasdermin D, resulting in membrane rupture and intracellular swelling by pore formation [27,32,33].

#### 2.1.6. Necroptosis

Necroptosis, a form of necrosis-like programmed cell death, is mediated by the receptor-interacting protein kinase-1 (RIPK1), -3 (RIPK3), and mixed lineage kinase domain-like protein (MLKL) [7,34]. This RIPK1-mediated necroptosis is initiated by TNF, Fas, TNFR1, and TLRs, followed by the formation of complex IIb (called ripotosome) composed of RIPK1 and pro-caspase-8. After that, necroptosome, composed of RIPK1, RIPK3, and MLKL, is assembled when caspase-8 is inactivated, and the following apoptosis is inhibited. Then, the activated MLKL can translocate to the cellular membranes, resulting in membrane disruption and cell lysis by pore formation [34,35]. 

#### 2.1.7. Neuronal Autophagy

Autophagy, which can be induced by the depletion of mitochondrial ATP, nutrients, ROS, and neurotrophic factors, is an essential process for the recycling of cellular components and damaged organelles [1,36]. In autophagy, phosphatidylinositol 3-phosphate (PI3P) is a key molecule in the formation of the autophagosome, followed by fusion with the lysosome and lysosomal degradation [36,37]. During neuroinflammation, excessive autophagy can lead to self-degeneration and the death of neuronal cells [1]. Recent studies have demonstrated that the neuronal autophagy process promoted the expression of pro-inflammatory molecules, thereby accelerating astrocyte death in oxidative stress via ROS [38,39]. 

#### 2.1.8. CNS Disorders with Neurodegeneration

Neuroinflammation-mediated neurodegeneration has been considered to play a key role in the pathogenesis of various CNS disorders such as AD, PD, HD, MS, ALS, cerebral ischemic diseases, and traumatic CNS injuries [2].

AD is a chronic and progressive neurodegenerative disease with cognitive and behavioral impairments. The distinctive features of AD are the overproduction and accumulation of Aβ plaques outside neuronal cells and the hyperphosphorylation and accumulation of tau tangles inside neuronal cells [18]. Both events contribute to neuronal cell death and progressive damages to the brain tissue. Several studies have reported that chronic neuroinflammation related to the over-activation of microglia and astrocytes can accelerate tau hyperphosphorylation and Aβ aggregation via the overproduction of pro-inflammatory cytokines [40,41,42]. PD is another chronic and progressive neurodegenerative disease with the continuous loss of dopaminergic neurons, thereby inducing motor, cognitive, and neuropsychiatric dysfunction. The distinctive features of PD are the accumulation of Lewy bodies and the overproduction, fibrillation, and accumulation of α-synuclein (αSN) [43]. Mitochondrial dysfunction, oxidative stress, and neuroinflammation can lead to neuronal degeneration in the *substantia nigra*, resulting in the aggregation of Lewy bodies and αSN and loss of dopamine [2,43]. A recent study demonstrated that BBB disruption and the subsequent infiltration of inflammatory mediators, including activated microglia, lead to dopaminergic neuronal cell death [44]. HD is also one of the neurodegenerative disorders and is caused by a genetic mutation in the huntingtin (*HTT*) gene on chromosome 4, resulting in the expression of an expanded trinucleotide repeat of the CAG sequence [45]. The resulting *HTT* proteins can lead to neuronal death. A recent study reported that the depletion of neurotrophic factors, such as brain-derived neurotrophic factor (BDNF), the overproduction of pro-inflammatory cytokines, the release of ROS, and glutamate-induced mitochondrial excitotoxicity, can accelerate neuronal damages in HD [46]. MS is a chronic, autoimmune disorder that causes the demyelination of the neuronal cells in the CNS tissues, resulting in axonal degeneration [2]. Mainly, BBB disruption and the following infiltration of immune cells such as T cells are involved in the pathogenesis of MS [47]. ALS is a progressive, neurodegenerative disease-causing damages in upper and lower motor neurons in CNS tissues. ALS can be induced by a point mutation in the superoxide dismutase 1 (SOD1) gene, and phosphorylated neurofilament (NF) and cystatin C can be identified in ALS [48]. Moreover, the activation of microglia and astrocytes, overproduction of pro-inflammatory cytokines, BBB disruption, and infiltration of T cells are associated with the pathogenesis of ALS [49]. 

Cerebral ischemic diseases, such as stroke, mainly induce the breakdown of the BBB and neurovascular damages as well as a lack of oxygen and nutrient supply in the brain [30,50]. Thus, BBB disruption, the subsequent infiltration of various pro-inflammatory mediators and immune cells, and hypoxia-induced ROS and NO production are involved in neuroinflammation processes on cerebral ischemic diseases [50,51]. In the case of traumatic CNS injuries, patients with traumatic brain injury (TBI) or spinal cord injury (SCI) experience loss of cognitive, sensory, and motor functions as well as chronic, long-lasting paralysis [52]. In primary injuries, acute necrotic neuronal cell death can be induced by the mechanical forces from various accidents on the CNS tissues (brain and spinal cord) [53]. The subsequent secondary injuries cause prolonged and chronic neuronal damages via various neuroinflammation processes, such as mitochondrial excitotoxicity, the release of ROS, axonal degeneration, and programmed cell death, resulting in the generation of lesional cavities and glial scars [54].

## 3. Neuroprotective Effects of Potential Natural Compounds and Their Limitation 

**Figure 2:**
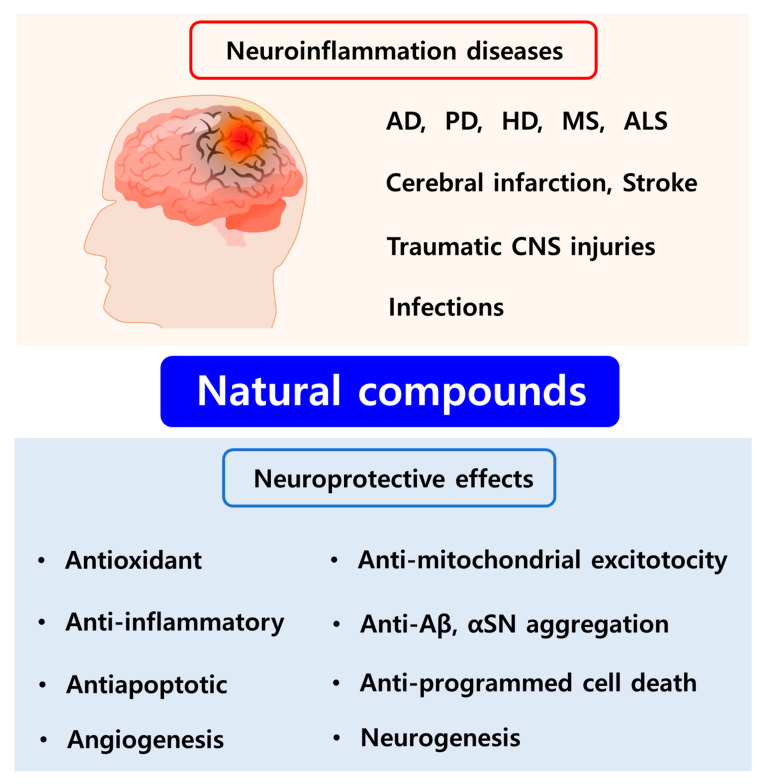
Various therapeutic effects of natural compounds in neuroinflammation. Alzheimer’s disease (AD); Parkinson’s disease (PD); Huntington’s disease (HD); Multiple sclerosis (MS); Amyotrophic lateral sclerosis (ALS); Central nervous system (CNS); Amyloid β protein (Aβ); α-synuclein (αSN).

**Figure 3:**
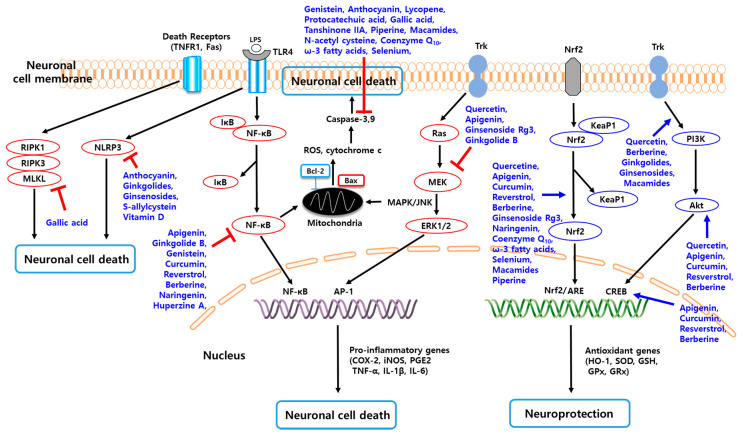
Molecular mechanisms of various natural compounds for the treatment of neuroinflammation.

Numerous studies have highlighted the capacity of natural compounds derived from plants, vegetables, fruits, dietary nutrients, and endogenous molecules to be used as potential therapeutics for the treatment of neuroinflammation mainly owing to their antioxidant, anti-inflammatory, antiapoptotic, and neuroprotective effects (Figure 2) [8,9,16]. Several studies have reported that many natural compounds can exhibit high affinity for various receptors in the brain, thereby specifically promoting or inhibiting various molecular signal transduction pathways and exerting multi-targeting effects on neuroinflammation-induced CNS disorders [55,56]. Another advantage of natural compounds is their lower side effects compared to conventional synthetic drugs [9,56]. Among the natural compounds, phytochemicals (e.g., flavonoid polyphenols, non-flavonoid polyphenols, phenolic acids, terpenoids, and alkaloids) and other dietary compounds have been extensively studied for the treatment of neuroinflammation-inducing CNS diseases including AD, PD, HD, MS, ALS, cerebral ischemia, and traumatic CNS injuries via various mechanisms of action (Figure 3) [8,55,56,57]. 

### 3.1. Various Natural Compounds for the Treatment of Neuroinflammation

#### 3.1.1. Flavonoid Polyphenols

Flavonoids are the most common natural compounds in plants, vegetables, fruits, and dietary foods. Flavonoids can exhibit antioxidant effects by inhibiting ROS production, anti-inflammatory effects by modulating various signaling pathways, and antiapoptotic effects by inhibiting proapoptotic molecules as well as angiogenic effects [58,59]. Apigenin, 4′,5,7-trihydroxyflavone, is a major constituent of chamomile herbs and can be synthesized from naringenin [60]. It exhibits various therapeutic effects including anti-inflammatory, anticancer, anti-allergic, cardioprotective, and neuroprotective efficacies [61]. Apigenin could protect BBB integrity and inhibit activation of the TLR4/NF-κB pathway, resulting in the suppression of neuronal cell apoptosis in a hemorrhage-induced brain injury model [62]. In a mouse AD model, apigenin promoted the production of neurotrophic factors and the activity of SOD and glutathione peroxidase (GPx) and prevented Aβ from fibrillation and aggregation by downregulating beta-site amyloid-protein precursor cleaving enzyme (beta-secretase-1; BACE-1) [63]. In addition, apigenin inhibited Ca^2+^ signaling by suppressing the activity of NMDAR, downregulated the production of pro-inflammatory mediators by inhibiting the p38 mitogen-activated protein kinase (MAPK) and stress-activated protein kinase (SAPK)/c-Jun N-terminal kinases (JNK) pathways, thereby inhibiting neuronal cell apoptosis in AD cell models [64].

Epigallocatechin-3-gallate (EGCG) is a major catechin in green tea and has antioxidant, anti-inflammatory, antitumor, and antimicrobial activities [65]. A study reported that EGCG reduced Aβ levels and plaque formation in transgenic mouse models of AD. In the same model, CD45, a marker of microglial activation, was reduced after EGCG treatment [66]. Another study demonstrated that EGCG inhibited the expression of pro-inflammatory cytokines, such as TNF-α, IL-1β, and IL-6, and the production of ROS, NO, and cyclooxygenase-2 (COX-2), thereby reducing Aβ levels and plaque formation in a lipopolysaccharide (LPS)-induced neuroinflammation model [67]. The beneficial effects of EGCG on the inhibition of Aβ aggregation in early AD patients are being studied in phase II/III trials (NCT00951834) [68].

Quercetin, 3,3′,4′,5,7-pentahydroxyflavanone, is one of the most powerful antioxidants of various edible plants. It exerts various therapeutic effects including anti-inflammatory, anticancer, anti-allergic, anti-infective, and neuroprotective efficacies [69]. Quercetin attenuated mitochondrial dysfunctions by inhibiting ROS production and increased the activity of SOD and ATP generation by activating the AMP-activated protein kinase (AMPK) pathway, thereby reducing Aβ-induced neurotoxicity [70]. In addition, quercetin inhibited neuroinflammation by suppressing microglia activation and pro-inflammatory mediator production via the inhibition of the NF-κB pathway and JNK phosphorylation in inflammation-induced microglial cells [71].

Naringenin, 4,5,7-trihydroxyflavanone, is one of the abundant flavonoids in citrus fruits and has antioxidant, anti-inflammatory, antiapoptotic, and neuroprotective effects [72]. A recent study reported that naringenin attenuated neurodegeneration by inhibiting ROS production, αSN aggregation, and pro-inflammatory mediator expression via the downregulation of the NF-κB pathway. In addition, naringenin reduced neuronal cell apoptosis by suppressing Bax proteins and increasing Bcl-2 proteins [73]. Moreover, naringenin promoted the suppressor of cytokine signaling 3 (SOCS-3) pathway and inhibited the MAPK and NF-κB pathways, thereby attenuating neuroinflammation in activated microglial cell models [74,75].

Genistein, 4′,5,7-trihydroxyisoflavone, is a major isoflavone found in soybean that acts similar to estrogen in the human body (phytoestrogen). It exerts various pharmacological activities including antioxidant, anti-inflammatory, angiogenic, anti-obesity, antiaging, and neuroprotective efficacies [76]. A study reported that genistein upregulated the phosphatidylinositol 3-kinase (PI3K)/Nrf-2 pathway, reducing oxidative stress, such as ROS, and promoting antioxidant enzyme activities [77]. In addition, genistein inhibited the TLR4/NF-κB pathway, thereby attenuating pro-inflammatory mediator expression and neuronal cell apoptosis [78]. Recently, genistein activated peroxisome proliferation-activated receptor γ (PPARγ) and promoted apolipoprotein E (ApoE) release, thereby accelerating Aβ clearance from the brain in an AD model [79]. 

Anthocyanins, found in a number of red and purple color-pigmented fruits and vegetables, have unique cationic structures and exhibit therapeutic effects on cardiovascular disease, cancer, and CNS disorders [80]. A recent study reported that anthocyanin-rich diets inhibited the production of ROS and pro-inflammatory mediators by upregulating the Nrf-2 pathway and downregulating the NF-κB pathway [81]. Another recent study demonstrated that anthocyanins had neuroprotective effects on an ischemic stroke model by suppressing NLRP3 expression and mitochondrial excitotoxicity, thereby inhibiting neuronal cell death [82].

#### 3.1.2. Non-Flavonoid Polyphenols

Curcumin, commonly found in *Curcuma longa*, is one of the oldest, traditional natural medicines exhibiting various beneficial effects including anti-inflammatory, antioxidant, immunomodulatory, anticancer, antidiabetes, cardiovascular-protective, and neuroprotective activities [83]. A recent study reported that curcumin promoted PPARγ activation and inhibited the NF-κB pathway, thereby suppressing the activation of microglia and the production of pro-inflammatory cytokines as well as inhibiting ROS production [84]. In addition, curcumin stimulated anti-inflammatory cytokine production and inhibited microglial activation by suppressing the JAK2/STAT3 pathway and promoting SOCS-1 expression, thereby attenuating plaque production [85]. Another recent study demonstrated that curcumin formulation based on solid lipids (Longvida^®^; Verdure Sciences, Inc., Noblesville, IN, USA), which has already been shown to improve its BA, suppressed the activation of microglia and astrocytes, resulting in improving the motor function of an neuroinflammation-induced mouse model [86]. The superior therapeutic effects of Longvida^®^ in AD patients are being studied in phase II trials (NCT01001637) [87].

Resveratrol, which is one of the famous non-flavonoid polyphenol ingredients found in grapes and red wine, exhibits various pharmacological effects such as cardioprotective, anti-inflammatory, antioxidant, antiaging, and neuroprotective activities [88]. Resveratrol protected neuronal cells from apoptotic death and inhibited reactive microglia-induced neuroinflammation by suppressing the MAPK/NF-κB pathway and activating sirtuin-1 (SIRT-1) [89,90]. Another study reported that resveratrol reduced the production of NO and ROS and enhanced the expression of hemoxygenease-1 (HO-1) through its antioxidant effects [91]. Recently, resveratrol reduced the production of pro-inflammatory mediators and promoted the production of anti-inflammatory factors in mild to moderate AD patients (phase II trials; NCT01504854), thereby attenuating Aβ_42_ and Aβ_40_ levels in CSF [92,93].

Lycopene, one of the most common carotenoids found in carrots, tomatoes, and papayas, exhibits various beneficial activities including antiproliferative, antitumor, antioxidant, anti-inflammatory, antidiabetes, cardiovascular-protective, and neuroprotective effects [94]. Lycopene inhibited pro-inflammatory mediator production and elevated antioxidant enzyme levels by upregulating the HO-1 activity and AMPK pathway and inhibited the apoptosis of neuronal cell by suppressing caspase-3 activity [95,96]. A recent study reported that lycopene suppressed the production of ROS, dysfunctions of mitochondria, and expression of Nucling, which promoted apoptosome assembly, by inhibiting the NF-κB pathway [97].

#### 3.1.3. Phenolic Acids

Natural phenolic acid compounds are generally derived from polyphenols by innate metabolisms. Protocatechuic acid, a phenolic metabolite of anthocyanins, exhibits various therapeutic effects including anti-inflammatory, antioxidant, antiapoptotic, anti-bacterial, antiviral, antitumor, and neuroprotective activities [98]. Protocatechuic acid promoted the activity of antioxidant enzymes and the expression of BDNF and inhibited the production of ROS, the release of glutamate, and the activation of caspase-3, thereby suppressing neuronal cell apoptosis in a hypoxia-induced neuroinflammation model [99]. A recent study reported that protocatechuic acid attenuated the release and expression of pro-inflammatory mediators by inhibiting p53 (a transcription factor of apoptosis) activation, upregulating SIRT1, and suppressing the NF-κB pathway [100].

Gallic acid, also known as 3,4,5-trihydroxybenzoic acid, is a phenolic acid compound found in the whole parts of many plants and has anti-inflammatory, antioxidant, antitumor, antiviral, and neuroprotective effects [101]. A recent study reported that gallic acid inhibited the production of ROS, disruption of mitochondrial membrane potential, and expression of Bax and caspase-3, and it promoted the expression of Bcl-2 and BDNF and upregulation of the Nrf-2 and cyclic adenosine monophosphate (cAMP) response element-binding protein (CREB) pathways, thereby protecting neuronal cells from neurodegeneration in a PD model [102]. Another study demonstrated that gallic acid also inhibited the necroptosis of neuronal cells by suppressing the expression of RIPK1 and RIPK3 [103].

#### 3.1.4. Terpenoids

Terpenoids are the most common and widespread secondary metabolites derived mainly from plants. Ginkgolides and bilobalide, major terpenoids from *Ginkgo biloba* extracts, exhibit various beneficial effects including anti-platelet-activating factor (PAF), anti-thrombosis, antioxidant, antiapoptotic, anti-inflammatory, and neuroprotective activities [104]. Ginkgolides suppressed the expression of NLRP3 and caspase-1 and inhibited -the nuclear translocation of NF-κB, thereby attenuating neuronal cell death [105]. In addition, ginkgolides not only reduced the production of pro-inflammatory mediators by downregulating the TLR4/NF-κB pathway but also promoted the inhibition of inflammatory M1 microglia and the elevation of anti-inflammatory M2 microglia in an ischemic stroke model [106]. Bilobalide also exhibited neuroprotective effects by upregulating the activities of antioxidant enzymes and downregulating the expression of ROS and pro-inflammatory cytokines via inhibition of the JNK/MAPK pathway in a cerebral ischemia model [107].

Tanshinone IIA, a lipophilic diterpene found in the Chinese traditional herb, Danshen, exhibits antioxidant, anti-inflammatory, antitumor, cardiovascular-protective, and neuroprotective effects. Tanshinone IIA reduced the production of pro-inflammatory mediators by inhibiting the NF-κB pathway, promoted the shift from M1 microglia to M2 microglia, and activated macrophage migration inhibitory factor (MIF), thereby attenuating neuroinflammation [108,109,110].

Ginsenosides, found in the *Panax ginseng* family, is one of the oldest, traditional natural medicines in East Asian countries, and it exhibits various beneficial effects including anti-inflammatory, antioxidant, immunomodulatory, anticancer, antidiabetes, antiaging, antidepression, anti-fatique, cardiovascular-protective, and neuroprotective activities [111]. Ginsenoside Rb2 and Re inhibited the production of pro-inflammatory mediators by suppressing the JNK phosphorylation and NF-κB pathway [112]. Ginsenoside Rg1 stimulated the expression of neurotrophic factors and attenuated neuronal cell apoptosis by upregulating the cAMP/protein kinase A (PKA)/cAMP response element binding protein (CREB) pathway [113]. In addition, a recent study reported that the neuroprotective effects of ginsenoside Rg1 were mediated by the upregulation of the Wnt/β-catenin signaling pathway [114]. In a clinical trial, ginsenoside Rd remarkably improved the disability scores and stroke scales in patients with acute ischemic stroke (phase III trials; NCT00815763) compared to the placebo group [115,116].

#### 3.1.5. Alkaloids

Alkaloids are natural-origin compounds containing carbon, hydrogen, and nitrogen (sometimes with oxygen). Berberine, an isoquinoline alkaloid found in the *Berberis* family, is also one of the oldest, traditional natural Chinese medicines exhibiting various beneficial effects including anti-inflammatory, antioxidant, anticancer, antidiabetes, anti-dyslipidemia, anti-fatigue, cardiovascular-protective, and neuroprotective activities [117]. Berberine inhibited the production of ROS and promoted the expression of antioxidant enzyems by upregulating the Nrf-2/HO-1 pathway. In addition, it attenuated neuronal cell apoptosis by suppressing the expression of Bax, cytochrome c, and caspases and upregulated Bcl-2 expression and the PI3K/Akt pathway [118]. Another study reported that berberine inhibited the production of pro-inflammatory mediators by downregulating the NF-κB and MAPK pathways in neuroinflammation-induced microglial cells [119].

Piperine, a major alkaloid found in *Piper nigrum* and *Piper longum*, exhibits various therapeutic effects including anti-inflammatory, antioxidant, antiapoptotic, gastroprotective, and neuroprotective activities [120]. Piperine stimulated antioxidant enzyme activity and inhibited various oxidative stresses and pro-inflammatory cytokine production. In addition, it attenuated neuronal cell apoptosis by suppressing the release of cytochrome c, expression of Bax, and caspases-3 and -9 activities and elevating the expression of Bcl-2 [121]. A recent study reported that piperine exerted neuroprotective effects by inhibiting the production of pro-inflammatory mediators via the downregulation of the NF-κB pathway and upregulation of the Nrf-2/HO-1 pathway [122].

Macamides, the non-polar and long-chain fatty acid structure-based alkamides, are found in Maca (*Lepidium meyenii* Walp.). These compounds recently demonstrated their antioxidant, anti-inflammatory, spermatogenetic, cognitive and memory function-improving, and neuroprotective effects [123]. Several studies have recently reported that macamides inhibited the production of oxidative stresses and promoted the activity of antioxidant enzymes in neuronal cells [124]. In addition, macamides attenuated neuroinflammation by preserving mitochondrial membrane potential, activating PPAR-γ, and suppressing the p38 MAPK pathway [125]. In addition, macamides exerted neuroprotective effects by inhibiting the expression of Bax, cytochrome c, cleaved caspase-3, and cleaved poly adenosine diphosphate (ADP)–ribose polymerase (PARP) and upregulating the production of BDNF and the CREB/Akt pathway [123].

#### 3.1.6. Other Dietary Compounds

Other dietary compounds are natural compounds not categorized in the above-mentioned groups and endogenous molecules needed by the body from food or as dietary supplements. S-allylcysteine, one of the most common organosulfur compounds found in garlic extracts, exhibits various beneficial effects including antioxidant, antiapoptotic, anti-inflammatory, immunomodulatory, and neuroprotective activities [126]. S-allylcysteine attenuated damages on neuronal cells by promoting the activities of antioxidant enzymes and suppressing lipid oxidation, NO synthase, and astrocyte activation [127]. A recent study reported that S-allylcysteine inhibited the production of inflammatory mediators by suppressing the TLR4/NF-κB pathway and microglia activation and upregulating the Nrf-2 pathway [128].

N-acetyl cysteine, a precursor of l-cysteine and glutathione (GSH) and naturally found in onion, is widely used as an antidote, antioxidant, and a dietary supplement. In addition, N-acetyl cysteine can exhibit anti-inflammatory, antiapoptotic, antiaging, and neuroprotective activities [129]. N-acetyl cysteine exerted neuroprotective effects in an HD model by inhibiting lipid peroxidation and ROS production and upregulating antioxidant enzyme expression. It also prevented mitochondrial dysfunctions, thereby inhibiting the release of cytochrome c and the expression of caspase-4 and p53 [130]. A recent study reported that N-acetyl cysteine prevented neuronal cell death from oxidative stress by promoting HO-1 activity [131]. In a recent clinical trial, N-acetyl cysteine significantly improved neurological functional outcomes and reduced various inflammatory biomarkers in patients with acute ischemic stroke (phase II trials; IRCT20150629022965N16) compared to the placebo group through its antioxidant and anti-inflammatory effects [132].

Vitamin D3 (cholecalciferol), considered as a steroid hormone, is a precursor of 1α,25-dihydroxy-vitamin D3 (calcitriol), which exhibits immunomodulatory, antioxidant, anti-inflammatory, and neuroprotective effects [133]. This vitamin D treatment attenuated the production of pro-inflammatory mediators by inhibiting TLR4 activity and promoted the shift from M1 microglia to M2 microglia [134]. A recent clinical study (phase II trials; IRCT20100407003655N4) demonstrated that vitamin D3 exerted neuroprotective effects in MS patients by upregulating the production and expression of anti-inflammatory cytokines and downregulating pro-inflammatory cytokines [135]. Another recent study reported that vitamin D3 also prevented BBB disruption and inhibited pyroptosis-mediated neuronal cell death and the expression of T cell transcription factors [136].

Coenzyme Q_10_ (ubiquinone), a benzoquinone compound, exists in our body tissues, plants, and animal organelles. It can exhibit antioxidant, anti-inflammatory, antiapoptotic, anticancer, antidiabetes, cardiovascular-protective, and neuroprotective effects [137]. A recent study reported that coenzyme Q_10_ inhibited lipid peroxidation, NO synthase, and ROS production and promoted the expression of antioxidant enzymes by upregulating the Nrf-2/HO-1 pathway. In addition, coenzyme Q_10_ inhibited the production of pro-inflammatory mediators and the expression of Bax and elevated the production of anti-inflammatory cytokines and the expression of Bcl-2 [138]. A clinical trial (phase IV trials; EudraCT200800744714) confirmed that coenzyme Q_10_ treatment in MS patients exerted the above-mentioned effects compared to without treatment with coenzyme Q_10_ [139]. In addition, it was recently demonstrated that coenzyme Q_10_ exerted neuroprotective effects mediated by enhanced angiogenesis and neurogenesis in a PD model [140].

ω-3 fatty acids such as eicosapentaenoic acid (EPA) and docosahexanoic acid (DHA), which are found mainly in fish oil, are one of the widely used polyunsaturated fatty acids as dietary supplements exhibiting anti-inflammatory, anti-plaque, anti-dyslipidemia, antihypertension, cognitive and memory function-improving, cardiovascular-protective, and neuroprotective effects [141]. A recent study demonstrated that ω-3 fatty acids inhibited pro-inflammatory mediator production, microglial activation, and neuronal cell apoptosis by suppressing the expression of high mobility group box 1 protein (HMGB1) and the TLR4/NF-κB pathway [142]. Another recent study reported that ω-3 fatty acids suppressed the activation of microglia and astrocytes, reduced Bax/Bcl-2 ratio, and promoted the production of BDNF, neuronal growth factor (NGF), and glial cell-derived neurotrophic factor (GDNF) by activating tyrosine kinase receptor B (TrkB). In addition, the authors proved that EPA exerted more powerful neuroprotective effects than DHA [143].

Se, one of the essential trace elements in humans, exists as selenocysteine or selenoproteins, which exhibited antioxidant and antidote activities against oxidative stress and xenobiotics [144]. Se inhibited the production of ROS, the release of cytochrome c, and activation of caspase-3 and -9 by suppressing the JNK/p38 MAPK pathway, thereby attenuating neuronal cell apoptosis in a traumatic brain injury model [145]. Another study reported that Se preserved mitochondrial membrane potential and respiratory activities by upregulating the expression of Nrf and PPARγ in a cerebral ischemia model [146]. In a recent study, Se reduced the production of pro-inflammatory mediators and the expression of PARP and upregulated the activities of antioxidant enzymes and the production of endogenous vitamin A and E, thereby attenuating neuronal cell death in a scopolamine-induced dementia model [147]. 

In summary, the potential natural compounds for the treatment of CNS inflammation and their mechanisms of action are presented in Table 1. Representative examples of natural compounds in the commercial market or clinical trials for the treatment of CNS inflammation are summarized in Table 2.

### 3.2. Physico-Chemical and Pharmacokinetic Limitations

Despite their numerous beneficial effects on neuroinflammation, the success of natural compound-based therapies is often impeded in vivo or in clinical use owing to their several limitations. First, natural compounds may have stability issues in the body (plasma instability) and during storage (pH, temperature, light, and humidity instability). Since most of them are antioxidants, they are sensitive to oxidative stress-inducing endogenous molecules, metal ions, and environments and/or endogenous catalytic enzymes [148,149]. The main challenge in the use of these unstable natural compounds as successful medicines for neuroinflammation is how to maintain their stability until they reach the target site. Second, given that non-invasive administration (per oral) can be more adequate than invasive, local administration via intracerebroventricular or intralesional injection in chronic CNS inflammatory diseases, most natural compounds are not easily acceptable for oral delivery owing to their poor aqueous solubility and poor oral absorption, thereby leading to poor systemic absorption and lower blood concentration [150]. Most poorly soluble natural compounds such as piperine are difficult to dissolve and diffuse in the gastrointestinal tract, resulting in poor oral absorption, whereas some hydrophilic natural compounds, such as ginsenoside Rg1 and Rb1 (Ginseng saponins), are unable to cross the epithelial cells of the intestine, resulting in the same consequence [151,152]. In addition, for the oral administration route, the acidic condition in the gastric and various enzymatic barriers and efflux pumps in the intestine hamper the systemic absorption of natural compounds [153,154]. Third, after the systemic absorption, rapid metabolism, and elimination of natural compounds (e.g., EGCG and curcumin) lead to lower levels in both blood and target tissue and reduction in the duration of their therapeutic action [155,156]. Lastly, limited distribution and localization into the target site, the brain across the BBB, are additional challenges to the development of natural compound-based therapy for clinical use in the treatment of neuroinflammation [17,157]. Tight junctions between endothelial cells of the BBB can restrict paracellular transport of large-molecular sized and hydrophilic natural compounds [158]. In addition, efflux transporters, such as P-glycoprotein (Pgp), breast cancer resistance protein (BCRP), and multidrug resistance-associated protein (MRP), on the surface of the BBB can pump out the transported natural compounds into the lumen of blood capillaries [27]. Thus, to circumvent the BBB, the intranasal delivery of natural compounds for the treatment of neuroinflammation has also been encouraged [159,160]. However, intranasal delivery also has limitations such as rapid mucociliary clearance, small administration volume, and undesired mucous toxicity [161].

In summary, the success of natural compound-based therapies is often hindered in their clinical use owing to their instability during storage or plasma instability, poor solubility and absorption, and/or poor BBB permeability, resulting in lower BA and lower distribution in the target tissue (brain), thereby failing to achieve the therapeutic concentration in the target tissue. To overcome these limitations, the role of various nanocarriers (e.g., polymeric nanoparticles (NPs), micelles, lipid NPs, liposomes, inorganic NPs, exosomes, and carbon-based NPs) via oral, intranasal, intravenous, or intraperitoneal administration routes has been emphasized continuously [83,150,162,163]. These nanocarriers can encapsulate or adsorb natural compounds efficiently, as a concept of nanomedicine, and improve their BA, transport across the BBB, and target the lesion sites in the brain, thereby enhancing their therapeutic efficacy for various neuroinflammation-induced CNS diseases. Thus, we introduce the various nanocarriers for the enhanced brain delivery of natural compounds as potential therapeutic strategies in the following paragraph.

## 4. Various Nanocarriers Containing Natural Compounds for the Treatment of Neuroinflammation

**Figure 4:**
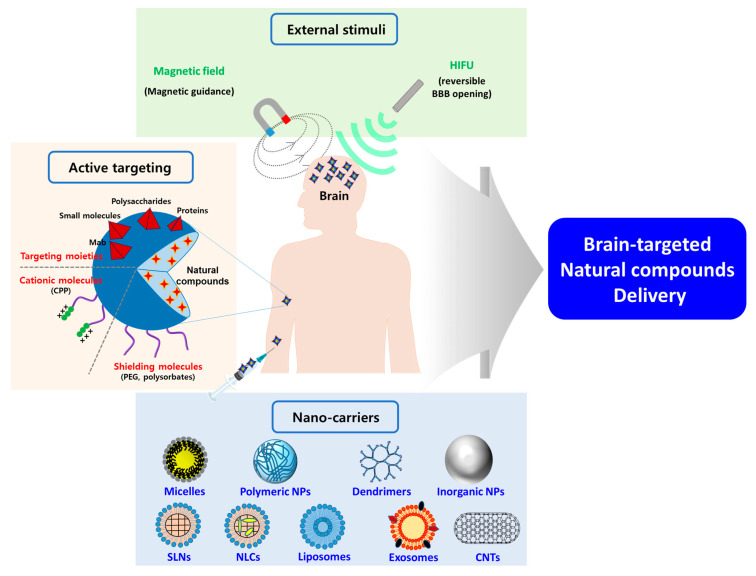
Schematic illustration of potential strategies using various nanocarriers for the enhanced brain delivery of natural compounds for treating neuroinflammation. Using various nanocarriers, surface functionalization with shielding moieties, cell-penetrating molecules, and targeting moieties, and applying external stimuli could be promising strategies for enhancing the delivery of natural compounds into the brain. High intensity focused ultrasound (HIFU); Blood–brain barrier (BBB); Cell-penetrating peptide (CPP); Polyethylene glycol (PEG); Nanoparticles (NPs); Solid lipid nanoparticles (SLNs); Nanostructured lipid carriers (NLCs); Carbon nanotubes (CNTs).

The utilization of nanocarriers as drug delivery systems has prominent advantages such as improved stability through encapsulation, enhanced solubility and permeability, controlled and prolonged drug release, enhanced BA, prolonged blood circulation, specific targeting, higher efficacy, and lower side effects [150,164,165,166]. To enhance the delivery of natural compounds into the brain, polymeric micelles, polymeric NPs, dendrimers, solid lipid NPs (SLNs), nanostructured lipid carriers (NLCs), liposomes, niosomes, carbon nanotubes (CNTs), Se NPs, metallic NPs, albumin NPs, and exosomes have been widely used with surface functionalization and external stimuli (Figure 4) [165,166,167].

### 4.1. Polymer-Based NPs

#### 4.1.1. Polymeric Micelles

Polymeric micelles are self-assembled nanocarriers using amphiphilic polymers that consist of hydrophobic domains as the inner core and hydrophilic domains as the outer shell with small particle size (10–100 nm) [168]. As a result of the existence of the hydrophobic inner core, lipophilic natural compounds can easily be encapsulated into the polymeric micelles to increase their solubility [168]. In addition, the hydrophilic outer shell, such as polyethylene glycol (PEG), allows the micelles to exhibit prolonged blood circulation, thereby enhancing the chance to reach the brain and intracellular uptake of encapsulated natural compounds [169]. A recent study of coenzyme Q_10_ delivery reported that PEG-α-tocopherol micelles enhanced the solubility and stability of encapsulated coenzyme Q_10_, improving its oral BA and brain accumulation, thereby enhancing neurological behavioral functions in a mouse PD model [170]. 

#### 4.1.2. Synthetic Polymer NPs

Among the most widely used synthetic polymers, polylactic acid (PLA), polyglycolic acid (PGA), and poly(lactic-co-glycolic acid) (PLGA) have been extensively studied for the enhanced drug delivery into the brain because of their ability to cross the BBB and exhibit surface functionalization with targeting moieties as well as their biodegradability and biocompatibility [171,172]. A study of tanshinone IIA delivery reported that cationic bovine serum albumin (CBSA)-conjugated PEG-PLA NPs enhanced systemic exposure of encapsulated tanshinone IIA, prolonged its blood circulation, and improved its brain accumulation, thereby exhibiting neuroprotective effects based on its anti-inflammatory and antiapoptotic activities in a rat ischemic stroke model [173]. The positively charged CBSA allowed the NPs to cross the BBB by electrostatic interaction with negatively charged endothelial cell membranes, which are also known as adsorptive mediated transcytosis [27,173]. Another recent study of ginsenoside Rg3 and thioflavin T delivery reported that angiopep-2-conjugated PLGA NPs allowed the encapsulated compounds to cross the BBB and reach glial cells, exhibiting neuroprotective effects based on its antioxidant, anti-inflammatory, antiapoptotic, and anti-Aβ plaque activities in an in vitro BBB model [174]. The angiopep-2 ligand can specifically bind to low-density lipoprotein receptor-related protein-1 (LRP-1) on the surface of the brain capillary endothelial cells, thereby facilitating cellular uptake across the BBB via receptor-mediated endocytosis [174,175].

Another alternative strategy for circumventing the BBB involves intranasal delivery. Natural compounds administered through this route can directly reach the cerebrospinal fluid (CSF) through the olfactory nerve and be efficiently delivered and accumulated in the CNS tissues [159,160]. However, owing to the above-mentioned limitations of intranasal delivery, the utilization of nanocarriers is an essential strategy for prolonged residence time and drug release in the nasal cavity [176]. A study of the delivery of urocortin peptide, a corticotropin endogenous molecule, reported that odorranalectin (OL)-conjugated PEG-PLGA NPs allowed urocortin to be highly accumulated in the brain tissue, thereby preventing dopaminergic neuronal cell death and enhancing neurological behavioral functions in a rat PD model [177]. The OL ligand can specifically bind to l-fucose on the surface of the nasal mucosa, enhancing mucoadhesion and improving the absorption of encapsulated natural compounds into the brain [177,178].

#### 4.1.3. Natural Polymer NPs

Natural polymer-based NPs have also been studied extensively for the enhanced drug delivery into the brain owing to their biodegradability and biocompatibility [179]. Among the various natural polymers, polysaccharide-based NPs have promising properties, such as biocompatibility, biodegradability, stabilization of entrapped molecules, controlled drug release, prolonged blood circulation time, easiness of surface functionalization, and their ability to target the BBB and brain cells for the treatment of neuroinflammation [180,181]. 

Among the polysaccharides (e.g., hyaluronic acid, chondroitin sulfate, and chitosan), chitosan (CS) NPs have been encouraged for intranasal administration owing to their positive-surface charge and mucoadhesive properties [182]. Naturally positive-charged CS can interact with the negatively charged mucosal epithelial cell membrane owing to its primary amine groups, thereby prolonging the residence time in the nasal cavity [183]. In addition, CS can bind to sialic acid (SA) in mucin, thereby loosening the tight junctions of the mucosal membrane and facilitating drug penetration through the olfactory nerve [184]. Moreover, CS itself can also exhibit neuroprotective effects based on its antioxidant activity that scavenges ROS [185]. Thus, CS NPs can be utilized as potential brain-targeted delivery systems via intranasal administration. A study of the intranasal delivery of piperine reported that triphosphate (TPP)-coated CS NPs exhibited a controlled release of piperine and enhanced its brain delivery by the mucoadhesive property of CS, exerting improved neuroprotective effects based on its anti-inflammatory and antiapoptotic acitivities in a rat AD model [186]. Another recent study of the intranasal delivery of huperzine A demonstrated that lactoferrin (Lf)-conjugated CS NPs allowed huperzine A to be more absorbed systemically and highly accumulated in the brain of rats by the mucoadhesive properties of CS and receptor-mediated endocytosis via the specific binding of the Lf ligand to Lf receptor, which was highly expressed on the BBB and brain cells [187]. 

#### 4.1.4. Dendrimers

Dendrimers, which are repeatedly branched polymers with generation (G), consist of a central core, interior layers, and terminal functional groups. Poly(amidoamine) (PAMAM), one of the most widely used dendrimers, can easily be surface-functionalized with various targeting moieties, which facilitates the translocation of dendrimers across the BBB [188]. Moreover, the positive surface charge of PAMAM dendrimers can promote cellular uptake by adsorptive mediated transcytosis and facilitate endolysosomal escape, thereby preventing loaded molecules from lysosomal degradation [189,190]. Particularly, a recent study demonstrated that hydroxyl group-modified G4 and G6 PAMAM dendrimers could be predominantly localized into activated microglial cells in an inflammation-induced rabbit kit model [191]. A previous study reported that N-acetyl cysteine-conjugated PAMAM dendrimers enhanced intracellular uptake and localization of N-acetyl cysteine, thereby elevating its antioxidant and anti-inflammatory activities in activated microglial cells [192].

### 4.2. Lipid-Based NPs

#### 4.2.1. Lipid NPs (Solid Lipid NPs; SLNs and Nanostructured Lipid Carriers; NLCs)

SLNs and NLCs are nano-sized (usually 100–400 nm) colloidal lipid particles consisting of solid lipids and surfactants with or without oils (liquid lipids). SLNs and NLCs have been encouraged for the brain delivery of natural compounds owing to their several advantages such as high loading capacity for hydrophobic molecules, improved stability, controlled drug release, and enhanced BA as well as biocompatibility [193,194]. Moreover, surface-conjugation with hydrophilic moieties such as PEG, poloxamers, and polysorbates and with targeting moieties such as ApoE in SLNs and NLCs allow prolonged blood circulation and targeted delivery across the BBB, respectively [195,196,197,198]. Particularly, SLNs and NLCs can be considered as promising nanocarriers for nose-to-brain delivery owing to the prolonged residence time in the nasal cavity by their occlusive effects and mucoadhesive properties [199]. A recent study of the intranasal delivery of astaxanthin reported that SLNs using poloxamer 188 as surfactant exhibited high drug-loading capacity and controlled drug release profiles and allowed astaxanthin to be highly accumulated in the brain of normal rats [200]. Based on these results, SLNs loaded with astaxanthin showed antioxidant activity in an oxidative stress-induced neuronal cell model. Another study of curcumin delivery demonstrated that Lf-conjugated PEGylated NLCs allowed the facilitation of curcumin transport across the BBB through receptor-mediated endocytosis, thereby exhibiting neuroprotection and attenuating neruronal cell damage based on its antioxidant activity in a rat AD model [201].

#### 4.2.2. Liposomes

Liposomes are conventional spherical vesicles consisting of a phospholipid bilayer. Liposomes have also been extensively studied for the enhanced drug delivery into the brain owing to their ability to load both hydrophilic and hydrophobic natural compounds with high drug-loading capacity, modify the surface charge, and functionalize with various targeting moieties as well as their biocompatibility [202,203]. To overcome the limitation of conventional liposomes, which can be easily removed by the endogenous reticuloendothelial system (RES) with rapid systemic clearance, several strategies have been exploited on liposomes, such as decreasing their particle size to under 100 nm, neutralizing their surface charge, and conjugating hydrophilic molecules (PEG or polysorbate) on the liposomal surface, thereby achieving more prolonged blood circulation [203,204]. A recent study reported that PEGylated and cationic liposomal formulations efficiently improved the BBB permeability of andrographolide [205]. Among the various functionalized liposomes, antibody-conjugated liposomes, also known as immunoliposomes, have been encouraged for brain-targeting delivery systems because they can facilitate BBB penetration and intracellular uptake via receptor-mediated endocytosis [206,207]. A recent study of curcumin delivery reported that transferrin receptor monoclonal antibody (TfR Mab)- and apolipoprotein E (ApoE)-conjugated liposomes allowed curcumin to be transported across the BBB and highly accumulated in the brain with its reduced distribution in other organs in a transgenic mouse AD model [208]. TfR Mab and ApoE ligands can be specifically recognized by TfR and LRP-1, respectively, which are highly expressed on the surface of the brain capillary endothelial cells; thus, they can accompany receptor-mediated endocytosis [208]. Therefore, the dual conjugation of specific targeting moieties on the surface of liposomes as a multifunctional concept is a promising strategy for overcoming the BBB [209]. 

### 4.3. Inorganic NPs

#### 4.3.1. Se NPs

Se NPs have been recently encouraged as a promising therapeutic strategy for the treatment of neuroinflammation owing to their own antioxidant activity, their ability to incorporate therapeutic molecules and conjugate various targeting moieties, and their ability to cross the BBB as well as low toxicity with biomaterial (i.e., polysaccharides) coating on neurons [210,211,212]. A recent study demonstrated that TfR Mab-PEG-conjugated Se NPs allowed Se to be highly accumulated and localized in the brain with its reduced distribution in other organs, thereby exhibiting neuroprotective effects by inhibiting the production of ROS and activation of the apoptosis process and regulating various signaling pathways in a rat ischemic stroke model [213]. Another recent study of sialic acid (SA) and Se delivery reported that peptide B6-coated SA-Se NPs allowed therapeutic molecules to be easily transported across the BBB and highly accumulated in glial cells, thereby exhibiting neuroprotective effects based on their antioxidant, antiapoptotic and anti-Aβ plaque activities in an in vitro BBB model [214].

#### 4.3.2. Gold NPs

Among the widely used metallic NPs, gold NPs have several advantages, such as simple preparation method, consistency of size distribution, high permeability through the BBB, easiness of surface functionalization, applicability for various imaging techniques (i.e., magnetic resonance imaging; MRI), and active targeting with external magnetic fields [215,216,217]. A recent study demonstrated that neuron-targeted rabies virus glycoprotein (RVG)-bound exosomes-coated gold NPs could be easily transported across the BBB and highly accumulated into the brain in an in vitro BBB model and normal rats [218]. Another recent study of anthocyanin delivery reported that PEG-coated gold NPs allowed anthocyanin to be highly accumulated in the brain across the BBB, thereby exhibiting enhanced neuroprotective effects based on its anti-inflammatory, antiapoptotic, and anti-Aβ plaque activities in a mouse AD model [219]. However, most metallic nanomaterials have a big challenge in clinical use because of their non-biodegradable properties and possible toxicity, especially in the brain tissues [220].

#### 4.3.3. Iron Oxide NPs

Iron oxide NPs, the most widely used magnetic NPs, are usually categorized as standard superparamagnetic iron oxide nanoparticles (SPIONs) depending on their particle sizes. SPIONs have several strengths as drug carriers, such as simple preparation method, easiness of surface functionalization and drug conjugation, applicability for MRI techniques, and relatively good biocompatibility [221]. In addition, SPIONs can penetrate the BBB more effectively in the presence of external magnetic forces or the application of high-intensity focused ultrasound (HIFU), thereby emerging as a promising theranostic strategy for CNS inflammation-induced diseases [222]. A recent study of the brain delivery of osmotin, a plant protein from tobacco, reported that dextran-coated SPIONs applied with an electromagnetic field allowed osmotin to be highly accumulated in the brain without any damages to the BBB, thereby efficiently attenuating Aβ accumulation and tau phosphorylation in a mouse AD model [223]. However, these iron oxide NPs still have safety concerns, especially in patients with neurodegenerative diseases, because of their ROS-inducing and Aβ-forming toxicity [224]. To overcome these safety concerns and reduce brain toxicity, biomaterial molecules, such as polysaccharides or hydrophilic polymers, have been exploited to be coated on iron oxide NPs [223,225]. 

### 4.4. Other NPs

#### 4.4.1. Carbon-Based NPs

Carbon nanotubes (CNTs), graphene or graphene oxide, and fullerene are a class of carbon-based nanocarriers. Among the carbon-based nanocarriers, CNTs have been the most widely utilized as drug delivery systems particularly for cancer and brain disorders owing to the easiness of surface functionalization with specific targeting moieties, thus enhancing their uptake in tumors, transport across the BBB, and their photothermal/photodynamic activities as well as high drug-loading capacity and controlled drug release [226,227]. In addition, coating hydrophilic polymers, such as polysorbates, or endogenous lipid, such as phospholipid, on the surface of CNTs can improve biocompatibility and reduce undesired toxicity [228]. In a previous study, amino group (NH_3_^+^)-functionalized CNTs penetrated the BBB and were delivered into the brain tissue in normal mice, which might be attributed to adsorptive mediated transcytosis that occurred by the positive surface charge of these CNTs [229]. Another recent study of berberine delivery reported that phospholipid- or polysorbate 80-coated CNTs exhibited sustained drug release and higher biocompatibility. In addition, both CNTs allowed berberine to be more absorbed systemically and highly accumulated in the brain across the BBB, thereby exhibiting its antioxidant efficacy and remarkably enhancing memorial function in a rat AD model [230]. Polysorbate 80, a P-gp blocking polymer, can be used as surface conjugation or surface coating to inhibit the efflux pump highly expressed on the BBB, improving drug accumulation in the brain tissue [231]. 

#### 4.4.2. Albumin NPs

Another promising type of NPs for enhanced brain delivery can be human serum albumin (HSA) NPs owing to their several strengths such as their ability to incorporate or bind with therapeutic molecules, controlled drug release, prolonged blood circulation, and easiness of surface functionalization with targeting moieties as well as non-toxicity, biodegradability, and biocompatibility [232,233]. A recent study of gallic acid delivery reported that the cationic polyethylenimine (PEI)-coated HSA NPs reduced neurodegeneration by inhibiting the fibrillation of αSN and suppressing the interaction between its oligomers and cell membrane. The enhanced neuroprotective effects of the HSA NPs might be attributed to the enhanced cellular uptake of gallic acid in the neuronal cells via adsorptive mediated transcytosis [234].

#### 4.4.3. Exosomes

Exosomes, which are derived from endogenous cells (i.e., stem cells), are nanovesicles whose particle size is 30–100 nm, and they can be used as novel nanocarriers for the enhanced brain delivery of natural compounds [235]. Since exosomes can be generated by almost all endogenous cells, they can exhibit good biocompatibility and interact with other cells in the target tissue [236]. Particularly, endogenous peptides, proteins, lipids, nucleic acids, and microRNAs, which exhibit neuroprotective effects, can be included in exosomes, resulting in a synergistic effect with the loaded therapeutic molecules [237]. In addition, exosomes can easily penetrate the BBB and be highly accumulated in the brain when they are surface-functionalized with specific targeting moieties [235]. Recent studies of natural compound (quercetin and curcumin) delivery reported that exosomes enhanced their BA, which was attributed by the improvement of solubility and stability and the prolongation of blood circulation. In addition, the exosomes allowed both natural compounds to be highly accumulated in the brain, which might be attributed to inherited endogenous targeting moieties bound on the surface of exosomes, thereby inhibiting tau hyperphosphorylation more efficiently in a mouse AD model [238,239]. 

In summary, various nanocarriers used for the enhanced brain delivery of natural compounds and their roles are presented in Table 3. 

### 4.5. Advanced NPs for Active Targeting of CNS Inflammation

To more efficiently improve the transport of natural compounds loaded into various NPs across the BBB and their accumulation in the brain, active brain-targeting methods based on specific binding between ligands and receptors, external magnetic field, or high-intensity focused ultrasound (HIFU) should be utilized [227]. In recent studies, advanced strategies for the active targeting of inflammatory cells or neurons have exploited specific targeting ligand functionalization, novel nanovalve systems, and special biomimetic systems, thereby enhancing the neuroprotective effects of loaded natural compounds. 

#### 4.5.1. Targeting Ligand-Functionalized NPs

The multifunctionalization of several targeting moieties or cationic cell penetration peptides on the surface of nanocarriers has been considered as a basic concept for active targeting of the brain [240]. Among the targeting moieties, specific ligands, which can bind to neutrophil, huntingtin protein, Aβ protein, oxidative stress-inducing H_2_O_2_, and reactive vascular endothelium, can be used to target CNS inflammation. The proline-glycine-proline peptide (PGP) ligand can specifically bind to CXCR2, which is highly expressed on infiltrating neutrophils. Thus, when using this ligand as targeting moieties with BBB-targeting ligands, such as T7 peptide or angiopep-2, the NPs loaded with natural compounds can be neutrophil-targeting systems toward the neuroinflammatory sites in the brain [241,242]. In the same way, trehalose ligands, which are multi-covalently linked to NPs, can specifically interact with intracellular huntingtin proteins in an HD-induced brain [243]. The Tet-1 peptide ligand can specifically interact with neurons, thereby localizing loaded natural compounds into the neuronal cells and enhancing their therapeutic efficacies [244]. The Arg-Gly-Asp-*D*-Tyr-Lys peptide (RGD) can specifically bind to integrin α_γ_β_3_, which is highly expressed on reactive vascular endothelial cells after ischemic stroke, thereby exhibiting specific targetability to the damaged cerebral blood capillary in a stroke-induce brain [245]. 

#### 4.5.2. Nanovalve Systems

In a recent study, novel nanovalve systems using host–guest interaction between β-cyclodextrin and ferrocene were exploited as a specific neuroinflammation-targeting strategy. In normal tissues, ferrocenes are located in the hydrophobic cavity inside β-cyclodextrin, thereby blocking the release of natural compound loaded into mesoporous NPs. However, in lesion sites damaged from oxidative stress, a high concentration of H_2_O_2_ changes ferrocenes (Fe atoms in the molecules) to oxidized ferrocenes (Fe^+^ ions in the molecules), which has a positive charge, resulting in the dissociation of the oxidized ferrocenes out of β-cyclodextrin, thereby initiating the release of the natural compound showing neuroprotective effects. Therefore, when using this β-cyclodextrin and ferrocene complex ligand as targeting moieties with BBB-targeting ligands, the NPs loaded with natural compounds can be oxidative stress (H_2_O_2_)-targeting systems toward the neuroinflammatory sites in the brain [246].

#### 4.5.3. Biomimetic NPs

Endogenous cell-, organelle-, or protein-biomimetic NPs can be promising nanocarriers for specific targeted delivery owing to their inherited properties, biocompatibility, and non-toxicity [247]. Particularly, endogenous cell-biomimetic NPs, also known as exosomes, can have their natural properties—inherited various endogenous molecules bound onto the bilayer of the biomimetic exosomes—and endogenous peptides, proteins, lipids, nucleic acids, and microRNAs included inside the exosomes. Owing to these features, biomimetic exosomes can exhibit specific targeting [248]. In a recent study, platelet membrane vesicles (PMVs) were exploited as a specific thrombus-targeting strategy for the treatment of ischemic stroke. PMVs, which have inherited targeting ligands to thrombus and nearby endothelial cells, can specifically bind to the thrombus [249]. These PMVs loaded with natural compound and magnetic NPs can reach the stroke-induced damaged sites faster and more active when external magnetic fields are applied near the damaged sites, because of the magnetic guidance effect. Thus, biomimetic exosomes can be promising nanocarriers exhibiting specific neuroinflammation-targeting delivery with the application of external magnetic field or HIFU or extra surface functionalization [249,250].

In summary, novel CNS inflammation-targeted strategies using nanocarriers containing natural compounds and their roles are presented in Table 4.

## 5. Conclusions and Future Remarks

Neuroinflammation, which is involved in various inflammatory cascades, occurs by microglia/astrocytes activation, oxidative stresses, and mitochondrial dysfunction in the nervous tissue, and it can result in persistent and chronic apoptotic neuronal cell death and programmed cell death (pyroptosis and necroptosis), thereby triggering various CNS degenerative disorders. The beneficial neuroprotective effects of natural compounds, derived from plants, vegetables, fruits, dietary nutrients, and endogenous molecules, against neuroinflammation are mainly mediated by their antioxidant, anti-inflammatory, and antiapoptotic effects with the specific promotion or inhibition of various molecular signal transduction pathways. However, although numerous natural compounds for CNS inflammation have been studied extensively in preclinical studies, few of them have successfully been developed as medicines. This is mainly because of their instability, poor solubility, and/or poor BBB permeability, resulting in lower BA, lower distribution in the target tissue (brain), and higher systemic toxicities.

To overcome these limitations and improve their pharmacokinetic properties and stability, various nanocarriers (e.g., polymeric NPs, micelles, lipid NPs, liposomes, inorganic NPs, exosomes, and carbon-based NPs) have been exploited, and these NPs loaded with natural compounds can improve BA, transport across the BBB, and targeting the lesion sites in the brain, thereby enhancing their therapeutic efficacy for various neuroinflammation-induced CNS diseases. Recently, promising CNS inflammation-targeted NP systems have been encouraged as lesion site-specific active targeting strategies. Specific targeting ligand-functionalized NPs, nanovalve systems, and biomimetic exosomes with the application of external magnetic field or HIFU have been developed for the neuroinflammation-specific delivery of natural compounds, thereby enhancing their therapeutic efficacy more efficiently.

However, there are still several limitations to the development of natural compound-based therapies as medicines. The issue of loading the natural compounds into the nanocarriers is a big challenge because it easily depends on the nature of the natural compounds and nanocarriers. Moreover, the problems of scale-up production of nanocarriers, the discrepancy between animal models and humans, insufficient information on the correlation between pharmacokinetics and pharmacodynamics in natural compounds, possible drug–drug interaction, and the complexity in pathophysiological microenvironments of neuroinflammatory diseases should be addressed before getting into the clinical trials. 

## Figures and Tables

**Table 1:** Potential natural compounds for the treatment of CNS inflammation.

Type of Natural Compounds	Therapeutic Agents	Mechanisms of Action	Ref.
Flavonoids	Apigenin	Direct radical scavenging action ↑, SOD ↑, GPx ↑, MDA ↓, ROS ↓, Ca^2+^ signaling ↓, NMDA receptor ↓, PKC ↓, BDNF ↑ Pro-inflammatory mediators (NO, iNOS, COX-2, IL-1β, IL-6, TNFα, GFAP) ↓, TLR4/NF-κB pathway ↓, p38 MAPK ↓, SAPK/JNK pathway ↓ Neuronal cell apoptosis ↓, caspase-3 and -7 ↓, cytochrome c ↓, Aβ levels ↓, BACE-1 ↓	[62,63,64]
	EGCG	ROS ↓, NO ↓, Nrf-2/ARE pathway ↑ Microglial activation ↓, iNOS ↓, COX-2 ↓, pro-inflammatory cytokines ↓, NF-κB pathway ↓, Aβ levels ↓, plaques formation ↓	[66,67]
	Quercetin	Neuroinflammation ↓, pro-inflammatory cytokines and proteins ↓, BACE-1 ↓, NF-κB ↓, αSN fibrillization ↓, Aβ ↓ Direct radical scavenging action ↑, ROS ↓, SOD ↑, AMPK ↑, Nrf-2/ARE ↑	[70,71]
	Naringenin	NO ↓, PGE2 ↓, iNOS ↓, COX-2 ↓, Pro-inflammatory cytokines and chemokine ↓, NF-κB ↓, AMPK ↑, SOCS3 pathways ↑ Nrf-2/ARE pathway ↑, ROS ↓, SOD ↑, GSH ↑, HO-1 ↑ Neuronal cell apoptosis ↓, cleaved caspase-3 ↓, Bax ↓, Bcl-2 ↑	[73,74,75]
	Genistein	ROS ↓, Nrf-2/HO-1 ↑, Inflammatory mediators (iNOS, COX, TNFα, and IL-1β) ↓, PPAR-γ ↑ Neuronal cell apoptosis ↓, cleaved caspase-3 ↓ Aβ ↓, plaque formation ↓, TLR4/NF-κB signaling pathway ↓	[77,78,79]
	Anthocyanins	Direct radical scavenging action ↑, intrinsic anti-oxidant (GSH, SOD, and Coenzyme Q10) ↑, Nrf-2 pathway ↑ Intracellular Ca^2+^ ↓, mitochondrial excitotoxicity ↓, Inflammatory mediators (iNOS, COX-2, TNFα, and IL-1β) ↓ JNK phosphorylation ↓, MAPK pathway ↓, NF-κB pathway ↓, inflammasome pathway (NLRP3) ↓ Neuronal cell apoptosis ↓, caspase-3 activity ↓, Bax ↓, Bcl-2 ↑	[81,82]
Non-flavonoid polyphenols	Curcumin	Direct radical scavenging action ↑, anti-oxidant proteins (CAT, GPx, SOD, HO-1, and GST) ↑, Nrf-2 pathway ↑ Inflammatory mediators ↓, pro-inflammatory cytokines ↓, anti-inflammatory cytokines ↑, PPAR-γ ↑, SOCS pathway ↑, NF-κB pathway ↓, STAT3 pathway ↓, Iba-1 (microglial activation) ↓, GFAP (astrocytes activation) ↓ Aβ ↓, plaques formation ↓, tau hyperphosphorylation ↓	[84,85,86]
	Resveratrol	Direct radical scavenging action ↑, GPx ↑, HO-1 ↑, NO ↓, ROS ↓, AMPK ↑ Pro-inflammatory factors (COX-1, COX-2, TNFα, NO) ↓, NF-κB pathway ↓, MAPK pathway ↓, SIRT-1 ↑ Programmed cell death ↓, Bax ↓, MMP-9 ↓, Aβ fibrillation and production ↓	[89,90,91]
	Lycopene	Direct radical scavenging action ↑, GPx ↑, GSH ↑, SOD ↑, HO-1 ↑, ROS ↓, NO ↓, Nrf-2 pathway ↑ Neuronal cell apoptosis ↓, caspase-3 ↓, Bax ↓, Bcl-2 ↑, Nucling (apoptosome complex) ↓, pro-inflammatory cytokines (TNFα, IL-1β, IL-6) ↓, iNOS ↓, NF-κB ↓, MAPK/JNK pathway ↓ BACE-1 ↓, Aβ ↓, tau phosphorylation ↓	[95,96,97]
Phenolic acids	Protocatechuic acid	Glutamate release ↓, direct radical scavenging action ↑, ROS ↓ Microglial activation ↓, pro-inflammatory mediators (NO, iNOS, COX-2, TNFα, IL-1β, IL-6, PGE2) ↓, BDNF ↑, SIRT-1 ↑, NF-κB pathway ↓, MAPK/JNK pathway ↓ Neuronal cell apoptosis ↓, cleaved caspase-3 ↓, p53 pathway ↓, Aβ fibrillation ↓, APP ↓	[99,100]
	Gallic acid	Direct radical scavenging action ↑, lipid peroxidation ↓, MDA ↓, SOD ↑, CAT ↑, GPx ↑, ROS ↓, Nrf-2 pathway ↑ CSPG ↓, GFAP ↓, ED-1 ↓, pro-inflammatory mediators (COX-2, NO, iNOS, IL-1β, TNFα) ↓, BDNF ↑, NF-κB pathway ↓ Bax ↓, Bcl-2 ↑, caspase-3 (apoptosis) ↓, RIPK-1 and RIPK-3 (necroptosis) ↓, Aβ and αSN aggregation ↓	[102,103]
Terpenoids	Terpenes in *Ginkgo biloba* extracts (Ginkgolides, Bilobalide)	MDA ↓, SOD ↑, GSH ↑, HO-1 ↑, ROS ↓, NO ↓, hippocampal Ca^2+^ ↓, Akt signaling ↑, Nrf-2 pathway ↑, BDNF ↑, BBB integrity ↑ Pro-inflammatory mediators (GFAP, MMP-9, iNOS, IL-1β, IL-6, TNFα) ↓, p38 MAPK ↓, TLR/NF-κB pathway ↓, PAF-signaling pathway ↓ Inflammatory M1 microglial cells ↓, anti-inflammatory M2 microglial cells ↑, NLRP3 inflammasome ↓, caspase-1 ↓ Neuronal cell apoptosis ↓, caspase-3,7,8,9 ↓, cytochrome c ↓, PARP ↓, Bax ↓, Bcl-2 ↑, PI3K/Akt pathway ↑, tau phosphorylation ↓	[105,106,107]
	Tanshinone IIA	Pro-inflammatory mediators (MMP-2, iNOS, PGE2, COX-2, IL-1β, IL-6, TNFα, MIF) ↓, NF-κB pathway ↓ MPO ↓, neutrophil infiltration ↓, M1 microglial genes ↓, M2 microglial genes ↑ Neuronal cell apoptosis ↓, Bcl-xL pathway ↑, Aβ levels ↓, BACE-1 ↓	[108,109,110]
	Ginsenosides	Direct radical scavenging action ↑, HO-1 ↑, SOD ↑, GPx ↑, MDA ↓, ROS ↓, Nrf-2 pathway ↑ cAMP/PKA/CREB pathway ↑, HIF-1α/VEGF pathway ↑, NSCs proliferation and differentiation ↑, BDNF ↑, IGF-1 ↑ Preservation of mitochondrial potential, PAR-1 ↓, BBB integrity ↑, immune cells infiltration ↓ NR2B ↓, glutamate signaling pathway ↓, glutamate- induced Ca^2+^ ↓, Iba-1 ↓, Pro-inflammatory mediators (GFAP, NO, iNOS, COX-2, IL-1β, IL-6, TNFα) ↓, NF-κB pathway ↓, STAT1 pathway ↓, p38 MAPK ↓, p-JNK ↓, PPAR-γ ↑ Neuronal cell apoptosis ↓, caspase-1, -3, -9 ↓, Bax ↓, Bcl-2 ↑, NLRP1 inflammasome ↓, Wnt signaling pathway ↑, PI3K/Akt pathway ↑ Aβ aggregation ↓, tau hyperphosphorylation ↓, BACE-1 ↓, αSN fibrillization ↓	[112,113,114]
Alkaloids	Berberine	MDA ↓, ROS ↓, SOD ↑, GSH ↑, HO-1 ↑, NMDA/glutamate signaling pathway ↓, Nrf-2 pathway ↑ Caspase-3, -9 ↓, cytochrome c ↓, Bax ↓, Bcl-2 ↑, PI3K/Akt pathway ↑ NGF ↑, cAMP/PKA/CREB pathway ↑, BBB integrity ↑, cerebral blood flow ↑ Pro-inflammatory mediators (NO, iNOS, COX-2, PGE2, IL-1β, IL-6, MCP-1, TNFα, TNFR1) ↓, NF-κB pathway ↓, p38 MAPK ↓, MAPK/ERK1/2 pathway ↓, AMPK pathway↑ Aβ accumulation and production ↓, APP ↓, BACE-1 ↓, tau phosphorylation ↓, GSK3 ↓	[118,119]
	Piperine	Lipid peroxidation ↓, MDA ↓, ROS ↓, SOD ↑, GSH ↑, HO-1 ↑, Nrf-2 pathway ↑ NMDA/glutamate signaling pathway ↓, BDNF ↑ Pro-inflammatory mediators (iNOS, COX-2, PGE2, IL-1β, IL-6, TNFα) ↓, NF-κB pathway ↓ Neuronal cell apoptosis ↓, caspase-3, -9 ↓, cytochrome c ↓, Bax ↓, Bcl-2 ↑, PARP ↓	[121,122]
	Macamides	FAAH inhibitors, AchE inhibitors MDA ↓, ROS ↓, SOD ↑, GSH ↑, GPx ↑, Preservation of mitochondrial potential, PPARγ ↑, BDNF ↑, cAMP/CREB pathway ↑ Neuronal cell apoptosis ↓, cleaved caspase-3 ↓, cytochrome c ↓, Bax ↓, Bcl-2 ↑, cleaved PARP ↓, PI3K/Akt pathway ↑	[123,124,125]
Other dietary compounds	S-allylcysteine	MDA ↓, ROS ↓, SOD ↑, CAT ↑, GSH ↑, HO-1 ↑, Nrf-2 pathway ↑ Pro-inflammatory mediators (GFAP, iNOS, IL-1β) ↓, TLR4/NF-κB pathway ↓, PPARγ ↑, Iba-1 ↓ Neuronal cell apoptosis ↓, NLRP1 and 3 inflammasome ↓	[127,128]
	N-acetyl cysteine	MDA ↓, ROS ↓, SOD ↑, GSH ↑, GPx ↑, HO-1 ↑, NMDA/glutamate signaling pathway ↓ Pro-inflammatory mediators (NO, iNOS, IL-1β, IL-6, TNFα, NSE, MMP-9) ↓, NF-κB pathway ↓, ICAM-1 ↓ Neuronal cell apoptosis ↓, caspase-3 ↓, cytochrome c ↓, p53 ↓, mitochondrial complex I ↑	[130,131,132]
	Vitamin D	Lipid peroxidation ↓, MDA ↓, ROS ↓, NGF ↑, GDNF ↑, NT3 ↑ Pro-inflammatory mediators (GFAP, iNOS, COX-2, IL-1β, IL-6, IL-17A, TNFα) ↓, anti-inflammatory cytokines (IL-4, IL-10, TGF-β) ↑, Iba-1 ↓, TLR4 ↓, SOCS3 pathways ↑, NLRP3 ↓, caspase-1 ↓, M1 microglia ↓, M2 microglia ↑	[134,135,136]
	Coenzyme Q_10_	Lipid peroxidation ↓, ROS ↓, SOD ↑, CAT ↑, GSH ↑, GPx ↑, HO-1 ↑, Nrf-2 pathway ↑ Laminin (angiogenesis) ↑, ATP ↑, glutamate ↓, GABA ↓, mitochondrial potential ↑ Pro-inflammatory mediators (NO, iNOS, IL-1β, TNFα) ↓, anti-inflammatory cytokines (IL-10) ↑, Neuronal cell apoptosis ↓, caspase-3 ↓, Bax ↓, Bcl-2 ↑, ubiquitin-proteasome ↓	[138,140]
	ω-3 fatty acids	AA ↓, Pro-inflammatory mediators (GFAP, iNOS, COX-2, PGE2, IL-1β, IL-6, TNFα, IFN-γ) ↓, HMGB1/TLR4/NF-κB pathway ↓, SIRT1 ↑, p38 MAPK ↓, PPARγ ↑, Iba-1 ↓ CD11b (microglia marker) ↓, APP ↓, PLA2 ↓, BDNF ↑, NGF ↑, GDNF ↑, TrkB (BDNF receptor) ↑ Neuronal cell apoptosis ↓, cleaved caspase-3 ↓, Bax ↓, Bcl-2 ↑, p75NTR ↓	[142,143]
	Se	ROS ↓, GSH ↑, GPx ↑, GDNF ↑, VEGF ↑, PPARγ ↑, Preservation of mitochondrial potential Pro-inflammatory mediators (IL-1β, TNFα) ↓, Ubiquitin-proteasome ↓, STAT3 pathway ↓, mTOR phosphorylation ↓, Wnt signaling pathway ↑, p38 MAPK ↓, SAPK/JNK pathway ↓ Neuronal cell apoptosis ↓, caspase-3, -9 ↓, cytochrome c ↓, Bax ↓, Bcl-2 ↑, Mst1 (pro-apoptotic kinase) ↓, PARP ↓	[145,146,147]

**↑** Upregulation of expression level, activity, or signaling pathway; **↓** Downregulation of expression level, activity, or signaling pathway. Central nervous system (CNS); Superoxide dismutase (SOD); Glutathione peroxidase (GPx); Malondialdehyde (MDA); Reactive oxygen species (ROS); N-methyl-D-aspartate (NMDA); Protein kinase C (PKC); Brain-derived neurotrophic factor (BDNF); Nitrogen oxide (NO); Inducible nitric oxide synthase (iNOS); Cyclooxygenase-2 (COX-2); Interleukin-1β (IL-1β); Tumor necrosis factor α (TNFα); Glial fibrillary acidic protein (GFAP); Toll-like receptor 4 (TLR4); Nuclear factor-kappa B (NF-κB); Mitogen-activated protein kinase (MAPK); Stress-activated protein kinase (SAPK); c-Jun N-terminal kinases (JNK); Amyloid β protein (Aβ); Beta-site amyloid-protein precursor (APP) cleaving enzyme (beta-secretase-1, BACE-1); Epigallocatechin-3-gallate (EGCG); Nuclear factor erythroid 2-related factor 2 (Nrf-2); Antioxidant response element (ARE); Adenosine monophosphate (AMP)-activated protein kinase (AMPK); α-synuclein (αSN); Prostaglandin E2 (PGE2); Suppressor of cytokine signaling 3 (SOCS3); Glutathione (GSH); Hemoxygenease-1 (HO-1); Peroxisome proliferation-activated receptor γ (PPARγ); Nucleotide-binding domain leucine-rich repeat and pyrin domain-containing protein 3 (NLRP3); Catalase (CAT); Glutathione S-transferase (GST); Signal transducer and activator of transcription 3 (STAT3); Ionized calcium-binding adapter molecule 1 (Iba-1); Sirtuin-1 (SIRT-1); Matrix metalloproteinase-9 (MMP-9); Amyloid-protein precursor (APP); Chondroitin sulfate proteoglycan (CSPG); Receptor-interacting protein kinase (RIPK); Platelet-activating factor (PAF); Poly adenosine diphosphate (ADP)–ribose polymerase (PARP); Phosphatidylinositol 3-kinase (PI3K); Macrophage migration inhibitory factor (MIF); Myeloperoxidase (MPO); Cyclic AMP (cAMP); Protein kinase A (PKA); cAMP response element binding protein (CREB); Hypoxia-inducible factor-1α (HIF-1α); Vascular endothelial growth factor (VEGF); Neural stem cell (NSC); Insulin-like growth factor 1 (IGF-1); Protease-activated receptor-1 (PAR-1); NMDA receptor 2B (NR2B); Neuronal growth factor (NGF); Tumor necrosis factor receptor 1 (TNFR1); Extracellular signal-related kinase 1 and 2 (ERK1/2); Glycogen synthase kinase 3 (GSK3); Glial cell-derived neurotrophic factor (GDNF); Neurotrophin 3 (NT3); Gamma-aminobutyric acid (GABA); Arachidonic acid (AA); High-mobility group box 1 (HMGB1); Phospholipase A2 (PLA2); Tyrosine kinase receptor B (TrkB); Neurotrophin receptor (NTR); Mammalian target of rapamycin (mTOR); Mammalian Ste20-like kinase 1 (Mst1).

**Table 2:** Natural compounds in the commercial market or clinical trials for the treatment of CNS inflammation.

Therapeutic Agents	Commercial Names /Clinical Phase	Distinctive Features	Type of Diseases	Ref.
EGCG	Phase II/III (NCT00951834)	Inhibition of amyloid aggregation	Early stage of AD	[68]
Curcumin	Longvida^®^, Phase II (NCT01001637)	Solid lipid formulation (higher BA and BBB penetration; half-life: 7.5 h)	Moderate to severe AD	[86,87]
Resveratrol	Phase II (NCT01504854)	Reduction of MMP-9, Aβ_42_ and Aβ_40_ levels in CSF, attenuation of pro-inflammatory cytokines (IL-1R4, IL-8, IL-12, TNF-α) production, and elevation of IL-4 and FGF-2 levels	Mild to moderate AD	[92,93]
Ginsenoside Rd	Phase III (NCT00815763)	Significant improvement in the disability scores and stroke scales compared to placebo group	Acute ischemic stroke	[115,116]
N-acetyl cysteine	Phase II (IRCT20150629022965N16)	Improvement of neurological functional outcomes, reduction of inflammatory biomarkers (IL-6, sICAM-1, NO, MDA, NSE), and elevation of antioxidant enzymes (SOD, GPx) levels by anti-oxidant and anti-inflammatory effects	Acute ischemic stroke	[132]
Vitamin D3	Phase III (IRCT20100407003655N4)	Downregulation of IL-17A expression and upregulation of TGF-β expression	MS	[135]
Coenzyme Q10 with IFN-β	Phase IV (EudraCT200800744714)	Reduction of pro-inflammatory mediator (IL-1β, IL-2R, IL-9, IL-17F, TNFα, IFN-γ, MIP-1α, GM-CSF) levels and elevation of anti-inflammatory cytokine (IL-4, IL-13) levels	MS	[139]

Central nervous system (CNS); Epigallocatechin-3-gallate (EGCG); Alzheimer’s disease (AD); Bioavailability (BA); Blood–brain barrier (BBB); Matrix metalloproteinase-9 (MMP-9); Amyloid β protein (Aβ); Cerebrospinal fluid (CSF); Interleukin (IL); Tumor necrosis factor-α (TNF-α); Fibroblast growth factor-2 (FGF-2); Soluble intercellular cell adhesion molecule-1 (sICAM-1); Nitric oxide (NO); Malondialdehyde (MDA); Neuron-specific enolase (NSE); Superoxide dismutase (SOD); Glutathione peroxidase (GPx); Transforming growth factor-beta (TGF-β); Interferon-beta (IFN- β);Multiple sclerosis (MS); Interferon-gamma (IFN-γ); Macrophage inflammatory proteins-1α (MIP-1α); Granulocyte-macrophage colony-stimulating factor (GM-CSF).

**Table 3:** Various nanocarriers for enhanced therapeutic effects of natural compounds on CNS inflammation.

Type of Nanocarriers	Nanocarriers (Administration Routes)	Therapeutic Agents	Role of Nanocarriers	Type of Diseases	Ref.
Polymer-based NPs	PEG-α-tocopherol micelles (oral)	Coenzyme Q_10_	The micelles solubilized hydrophobic coenzyme Q_10_ and enhanced its stability. The micelles improved its BA and delivery to brain.	MPTP-induced mouse model of PD	[170]
	CBSA-conjugated PEG-PLA NPs (intravenous)	Tanshinone IIA	Positive charge of CBSA allowed tanshinone IIA to be more accumulated to the brain tissue through adsorptive mediated transcytosis. The NPs improved drug exposure and prolonged blood circulation.	MCAO surgery-induced rat cerebral ischemic stroke model	[173]
	Angiopep-2-conjugated PLGA NPs	Rg3 and thioflavin T	Angiopep-2 ligand allowed the NPs to cross the BBB and reach glial cells. Thioflavin T, encapsulated into the NPs, exhibited targeting Aβ fibrils.	In vitro BBB model using Aβ_1-42_ -pretreated C6 glial cells	[174]
	OL-conjugated PEG-PLGA NPs (intranasal)	Urocortin	OL ligand allowed the NPs to be more accumulated to the brain by its mucoadhesive properties and specific binding to l-fucose expressed on the olfactory epithelium.	6-OHDA-induced rat model of PD	[177]
	TPP-CS NPs (intranasal)	Piperine	Positive charge of CS can exhibit absorption-enhancing effect and mucoadhesive properties, thereby improving nose-to-brain delivery of piperine. Negative charge of TPP allowed high loading efficiency of piperine.	Colchicine-induced rat model of AD	[186]
	Lf-conjugated TMCS NPs (intranasal)	Huperzine A	Positive charge of TMCS can exhibit absorption-enhancing effect and mucoadhesive properties. Lf ligand facilitated transportation into the brain through receptor-mediated endocytosis. The NPs improved absorption and brain distribution of huperzine A.	KM mouse (model for age-related decline)	[187]
	Anionic PAMAM dendrimers	N-acetyl cysteine (conjugated with dendrimer)	The dendrimers rapidly entered the neuronal cells and localized in the cytoplasm despite of their anionic charge. Based on this enhanced intracellular uptake, anti-oxidant and anti-inflammatory effects of drug were improved.	LPS-induced neuroinflammation in BV-2 cells	[192]
Lipid-based NPs	SLNs (intranasal)	Astaxanthin	SLNs showed high drug-loading capacity and controlled release patterns. SLNs enhanced localization of astaxanthin in the brain.	H_2_O_2_-induced neurodegeneration in PC12 cells	[200]
	Lf-conjugated NLCs (intravenous)	Curcumin	Lf ligand facilitated transportation across the BBB through receptor-mediated endocytosis, resulting in higher accumulation and localization of curcumin into the brain with reduced systemic distribution.	Aβ_1-42_- and D-gal- induced rat model of AD	[201]
	TfR Mab- and ApoE-conjugated liposomes (intravenous)	Curcumin derivative (as lipid component)	Dual, BBB specific ligands transported liposomes across the BBB. Lipid-derivative of curcumin allowed the liposomes to be targeted to amyloid peptides in the brain.	APP/PS1 transgenic mouse model of AD	[208]
Inorganic NPs	Peptide B6-coated SA-Se NPs	SA and Se	Peptide B6 ligand allowed the NPs to be more uptake into the brain tissue (as PC12 cells) across the BBB (as bEND.3 cells).	In vitro BBB model using Aβ monomer -pretreated bEND.3 cells and PC12 cells	[214]
	PEG-coated gold NPs (intravenous)	Anthocyanin	The NPs allowed anthocyanin to be highly accumulated into the brain across the BBB without cytotoxic effect.	Aβ_1-42_-induced mouse model of AD	[219]
	Dextran-coated SPIONs (intravenous)	Osmotin	Dextran coating can diminish undesired brain toxicity of SPIONs. Application of external magnetic field allowed the SPIONs and osmotin to be accumulated into the brain specifically (magnetic targeting) without disrupting the BBB integrity.	Aβ_1-42_-induced mouse model of AD	[223]
Carbon-based NPs	PL- and polysorbate 80-coated MWCNTs (intravenous)	Berberine	MWCNTs led to sustained release of berberine. PL and polysorbate 80 coating let to higher biocompatibility of MWCNTs. The MWCNTs allowed berberine to be more transported into neuronal cells, be more absorbed systemically, and be accumulated in the brain across the BBB.	Aβ-induced rat model of AD	[230]
Biomimetic NPs	PEI-coated HSA NPs	Gallic acid	The cationic NPs reduced neurodegeneration by inhibiting fibrillation of αSN and interaction between its oligomers and cell membrane. It might be attributed to enhanced drug transportation into the neuronal cells via adsorptive mediated transcytosis.	αSN aggregates-treated PC12 cells (HD model)	[234]
	Exosomes (intraperitoneal)	Quercetin, Curcumin	Exosomes enhanced drug BA owing to improved solubility and stability and prolonged half-life. Exosomes accelerated drug accumulation into the brain owing to their inherited targeting moieties.	OA-induced mouse model of AD	[238,239]

Central nervous system (CNS); Polyethyleneglycol (PEG); Bioavailability (BA); 1-Methyl-4-phenyl-1,2,3,6-tetrahydropyrine (MPTP); Parkinson’s disease (PD); Poly-l-lactide (PLA); Nanoparticle (NP); Cationic bovine serum albumin (CBSA); Middle cerebral artery occlusion (MCAO); Poly(lactic-*co*-glycolic acid) (PLGA); Odorranalectin (OL); 6-Hydroxydopamine (6-OHDA); Blood–brain barrier (BBB); Amyloid β protein (1-42) (Aβ_1-42_); Ginsenoside Rg3 (Rg3); Polyamidoamine (PAMAM); Lipopolysaccharides (LPS); Mouse microglial cell line (BV-2); Lactoferrin (Lf); N-trimethylated chitosan (TMCS); Kunming (KM); Tripolyphosphate (TPP); Chitosan (CS); Alzheimer’s disease (AD); Solid lipid nanoparticle (SLN); Kainic acid (KA); Pheochromocytoma cell line (PC12); Nanostructured lipid carrier (NLC); D-galactose (D-gal); Transferrin receptor monoclonal antibody (TfR Mab); Apolipoprotein E (ApoE); Phospholipid (PL); Multi-walled carbon nanotube (MWCNT); Sialic acid (SA); Selenium (Se); Brain capillary endothelial cell (bEND.3); Superparamagnetic iron oxide nanoparticle (SPION); Polyethyleneimine (PEI); Human serum albumin (HSA); α-synuclein (αSN); Huntington’s disease (HD); Okadaic acid (OA).

**Table 4:** Novel CNS inflammation-targeted strategies using nanocarriers containing natural compounds.

Targeting Strategies	Nanocarriers	Therapeutic Agents	Role of Nanocarriers and Observed Effects	Type of Diseases	Ref.
Dual-ligand functionalization (Neutrophil-targeting)	T7- and PGP-conjugated PEG-PAMAM dendrimers, Angiopep-2- and PGP-conjugated PEG-PAMAM dendrimers	Tanshinone IIA, Scutellarin	Dual-targeting ligands transported the nanocarrier across the BBB and targeted neutrophil, resulting in enhancing drug accumulation in the brain. The dendrimer NPs suppressed the concentration of intracellular Ca^2+^ and production of pro-inflammatory cytokines by inhibiting the HMGB1/TLRs signaling pathway.	MCAO surgery-induced rat cerebral ischemic stroke model	[241,242]
Natural compounds functionalization (Huntingtin-targeting)	Iron oxide-corded zwitter ionic polyacrylate NSs	Trehalose (covalently linked with NSs)	Zwitter ionic NSs can interact with cell membrane, thereby crossing the BBB. Multiple terminal trehalose offered interaction with intracellular huntingtin peptides, which leads to enhanced brain targeting. The NSs efficiently inhibited mutant huntingtin aggregation and amyloid fibrillation, resulting in attenuation of neurodegeneration.	Mouse model of HD	[243]
Natural compounds functionalization (Aβ- and neuron- targeting)	Tet-1-coated EGCG-Se NPs	EGCG (conjugated on Se core)	Tet-1 and EGCG ligands allowed the NPs to specifically interact with neurons and Aβ, respectively, resulting in enhancement of brain delivery. The NPs improved inhibition of EGCG on Aβ aggregation and ROS production, thereby attenuating apoptotic response.	Aβ monomer or disaggregated Aβ fibrill -pretreated PC12 cells	[244]
Ligand-functionalized exosome (Reactive vascular endothelium-targeting)	Cyclic RGDyK-conjugated exosomes	Curcumin	The ligand conjugation enhanced specific uptake of exosomes into reactive endothelial cells and the exosomes migrated to the lesion region of the ischemic brain. The exosomes reduced production of pro-inflammatory cytokines and expression of cleaved caspase-3 and attenuated activation of microglia and NF-κB pathway.	MCAO surgery-induced mouse cerebral ischemic stroke model	[245]
β-CD nanovalves (H_2_O_2_ and Aβ- targeting)	Bor-β-CD/Fc complexes-conjugated MSe NPs	Resveratrol	Bor ligand can interact with cell membrane, thereby allowing the NPs to cross the BBB. β-CD/Fc exhibited H_2_O_2_-sensitive dissociation, followed by release of resveratrol into Aβ-induced lesion site. The NPs improved drug BA and prolonged its blood circulation. The NPs attenuated neuroinflammation via the downregulation of pro-inflammatory cytokines, NO, and ROS and upregulation of anti-inflammatory cytokines. The NPs inhibited the formation of Aβ and tau phosphorylation, thereby attenuating neuronal cell death.	APP/PS1 transgenic mouse model of AD	[246]
Biomimetic magnetic NP (Thrombus-targeting and magnetic guidance)	Iron oxide NPs-loaded PMVs	l-arginine	PMVs can recognize damaged blood vessels and specifically bind to thrombus in the lesion site of the ischemic brain. Application of an external magnetic field allowed the PMVs to be more quickly adhered and more accumulated in the lesion site owing to loaded iron oxide NPs. The PMVs modulated the production of NO, resulting in promotion of revascularization. The PMVs enhanced the expression of CD31, leading to the attenuation of prolonged inflammatory response.	Focal cerebral ischemia mouse model	[249]

Central nervous system (CNS); Poly(lactic-*co*-glycolic acid) (PLGA); Polyethyleneglycol (PEG); High lipophilic triphenylphosphonium (L5 TPP); Blood–brain barrier (BBB); Superoxide dismutase 1 (SOD1); HAIYPRH peptide (His-Ala-Ile-Tyr-Pro-Arg-His; T7); Proline-glycine-proline (PGP); Polyamidoamine (PAMAM); Nanoparticle (NP); High mobility group box 1 protein (HMGB1); Toll-like receptor (TLR); Middle cerebral artery occlusion (MCAO); Nanoshell (NS); Huntington’s disease (HD); Amyloid β protein (Aβ); HLNILSTLWKYR peptide (Tet-1); Epigallocatechin-3-gallate (EGCG); Selenium (Se); Reactive oxygen species (ROS); Pheochromocytoma cell line (PC12); β-cyclodextrin (β-CD); Borneol (Bor); Ferrocene (Fc); Bioavailability (BA); Nitrogen oxide (NO); Reactive oxygen species (ROS); Mesoporous selenium (MSe); Alzheimer’s disease (AD); Arg-Gly-Asp-D-Tyr-Lys peptide (RGDyK); Nuclear factor-kappa B (NF-κB); Platelet membrane vesicle (PMV).
